# ﻿*Orychophragmus yangii* sp. nov. (Brassicaceae), a new species from Shaanxi, China

**DOI:** 10.3897/phytokeys.262.164767

**Published:** 2025-09-05

**Authors:** Lu-Lu Xun, Yong-Xing Qin, Pei-Liang Liu, Ming Yue, Bin Li, Yong-Fu Chai

**Affiliations:** 1 Xi’an Botanical Garden of Shaanxi Province (Institute of Botany of Shaanxi Province), Xi’an, Shaanxi 710061, China Xi’an Botanical Garden of Shaanxi Province (Institute of Botany of Shaanxi Province) Xi’an China; 2 Key Laboratory of Resource Biology and Biotechnology in Western China, Ministry of Education, Northwest University, Xi’an, Shaanxi 710069, China Northwest University Xi’an China

**Keywords:** Loess Plateau, morphological characters, *
Orychophragmus
*, phylogenetic position

## Abstract

A new species, *Orychophragmus yangii***sp. nov.**, from the Loess Plateau of Shaanxi Province, China, is described and illustrated. This species differs clearly from all other *Orychophragmus* species in the following traits: whole plant glabrous; leaves varying from simple to pinnatifid but never lyrate; pinnatifid leaves with lobes divided or not; simple leaves widest at the base; sepals green; petals white; fruit narrowly linear, terete, and glabrous; rostra of fruit 2–4 mm long. Molecular phylogenetic analyses based on plastid genomic data and nuclear ribosomal RNA genes showed that the new species represents the first divergent lineage in the genus *Orychophragmus*. The chromosome number of the new species is 2*n* = 24.

## ﻿Introduction

*Orychophragmus* Bunge is a genus of the family Brassicaceae, with species mainly distributed in China. *O. violaceus* (L.) O. E. Schulz also extends to Korea (Zhou et al. 2001). For a long time, several species of this genus were merged into *O. violaceus* (Al-Shehbaz 2000). However, studies on the morphology of seedlings, leaves, flowers, pollen, fruits, and seeds do not support the merger (Zhang et al. 2005; Zhang et al. 2009; Chen et al. 2017). Moreover, *Cardamine limprichtiana* Pax and *Alliaria grandifolia* C. H. An were merged and transferred to this genus as *O. limprichtianus* (Pax) Al-Shehbaz & G. Yang (Zhou et al. 2001; Wang et al. 2012). Later, *O. limprichtianus* was transferred to a new genus as *Sinalliaria limprichtiana* (Pax) X. F. Jin et al. (Zhou et al. 2014).

Molecular phylogenetic evidence was used to delimit species in *Orychophragmus*, and seven species were confirmed, including two newly published species, *O. zhongtiaoshanus* Huan Hu et al. and *O. longisiliquus* Huan Hu et al. (Zhou et al. 2009; Hu et al. 2015, 2016, 2018). Phylogenetic analyses also confirmed that the genus *Sinalliaria* X. F. Jin et al. is sister to *Orychophragmus*.

The Loess Plateau is located in the middle and upper reaches of the Yellow River in northwestern China and is generally covered with deep loess soil. From the southeast to the northwest of the Plateau, the climate changes gradually from sub-humid to semi-arid and arid (Wang et al. 1991). The Loess Plateau is relatively rich in plant species, with 3,224 species of seed plants (Zhang et al. 2002).

We first learned about a putative new species of *Orychophragmus* from Mr. Hong-Hua Yang, a traditional Chinese physician, who noticed it in 1976. Subsequently, specimens of *Orychophragmus* deposited in WUK, PE, HIB, and XBGH were examined, and specimens of this putative new species were repeatedly collected from Suide and Wubu counties in Shaanxi Province in the Loess Plateau. Our detailed morphological observations and molecular phylogenetic analyses confirmed it as a new species, which is reported herein.

## ﻿Materials and methods

### ﻿Taxon sampling

Two accessions of the new species and six species of *Orychophragmus* were included in the phylogenetic analyses. *O. ziguiensis* was not included because of its extinction (Hu et al. 2016), and a previous phylogenetic study showed that it was sister to *O. hupehensis* (Pamp.) Z. M. Tan & X. Liang Zhang (Hu et al. 2015). *Sinalliaria* and several other genera in Brassicaceae were used as outgroups (Hu et al. 2015, 2016) to determine the position of the new species. The new species and *O. taibaiensis* Z. M. Tan & B. X. Zhao were sequenced in this study, while sequences of the other taxa were acquired from GenBank (www.ncbi.nlm.nih.gov). Taxon names, voucher information of the newly sequenced samples, and GenBank accession numbers are provided in Appendix 1.

### ﻿DNA sequencing, assembly, and annotation

Leaf materials were collected from the field and dried in silica gel. Leaf samples were sent to Biomarker Technologies (Beijing, China) for total genomic DNA extraction, library preparation, and Illumina paired-end sequencing. *De novo* assemblies of the plastid genome and the nuclear ribosomal RNA genes 18S, ITS1, 5.8S, ITS2, and 26S were performed using GetOrganelle v.1.7.6.1 (Jin et al. 2020). Annotation of the plastid genes was completed on the CPGAVAS2 online platform (Shi et al. 2019) with *O. violaceus* (KX364399) as the reference. The nuclear genes were annotated in Geneious v.9 (Kearse et al. 2012) with *Brassica napus* L. (KX709366) as the reference.

### ﻿Phylogenetic analyses

Sequences were aligned using the MAFFT plug-in in Geneious v.9. The best-fit nucleotide substitution model was determined by ModelFinder (Kalyaanamoorthy et al. 2017). The GTR+F+I+G4 model was applied to both the nuclear and plastid datasets. Maximum likelihood (ML), maximum parsimony (MP), and Bayesian inference (BI) analyses were conducted using IQ-TREE v.1.6.7 (Nguyen et al. 2015), PAUP* 3.99.169 (Swofford 2002), and MrBayes v.3.2.6 (Ronquist and Huelsenbeck 2003; Ronquist et al. 2012), respectively. Posterior probabilities (PP) from the BI and bootstrap support percentages from the ML and MP analyses (BSML and BSMP) were labeled on the corresponding branches of the BI trees.

### ﻿Chromosome number count

Seeds of the new species were collected from two populations and germinated in a glass dish with moist filter paper at room temperature. When the roots grew to about 5 mm long, they were treated in 2 mmol·L^−1^ 8-hydroxyquinoline solution at room temperature for 4 H, then soaked in Carnoy’s solution (ethanol:glacial acetic acid = 3:1) overnight at 4°C. The roots were dissociated with 1 Mol·L^−1^ hydrochloric acid at 60°C for 2.5 Min and rinsed thoroughly with tap water. The root tips were stained with carbol fuchsin for about 15 Min and squashed on a glass slide. Well-spread mitotic metaphase chromosomes were examined and photographed using a 100× oil lens on a Nikon Eclipse 55i microscope.

## ﻿Results

### ﻿Plastid data

**Figure 1.**
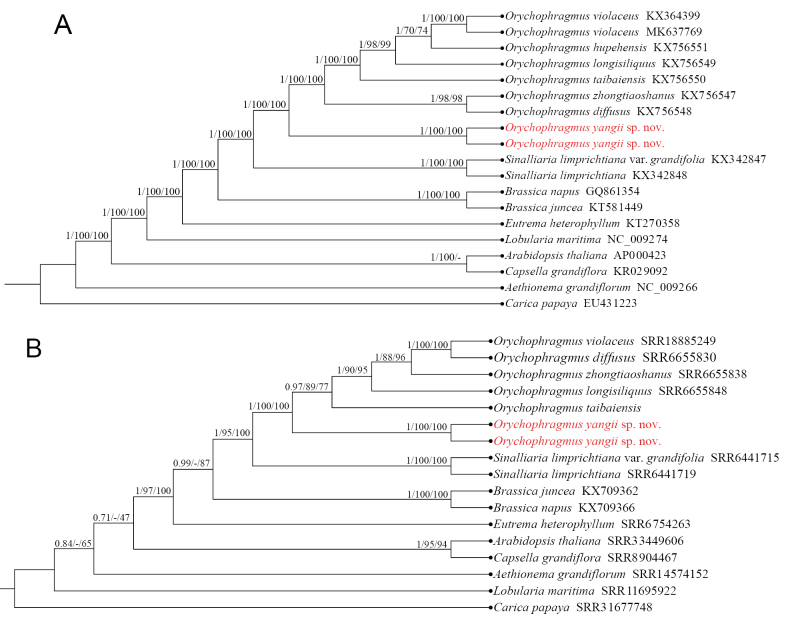
Bayesian tree based on the plastid genomic sequences (A) and the nuclear ribosomal RNA genes 18S, ITS1, 5.8S, ITS2, and 26S sequences (B), showing the phylogenetic position of *Orychophragmus yangii*. The Bayesian posterior probabilities (PP) and the percentages of bootstrap of the MP and ML (BSMP, BSML) are given on each branch. The dash (–) indicates that the clade is not supported in the MP tree.

The phylogenetic tree based on the plastid genomic data (Fig. 1A) shows that the new species is the first divergent lineage in the genus *Orychophragmus*, with high support values (PP = 1; BSMP = 100%; BSML = 100%). The relationships among the other six species of *Orychophragmus* are congruent with the previous plastid tree (Hu et al. 2016).

### ﻿Nuclear data

The nuclear tree (Fig. 1B) also shows that the new species is the most basal lineage in the genus *Orychophragmus* with high support values (PP = 1; BSMP = 100%; BSML = 100%). The relationship among the other five species of *Orychophragmus*, however, is different from the plastid tree. This may be a result of incomplete lineage sorting and/or hybrid introgressions as discussed by Hu et al. (2016).

### ﻿Chromosome number count

**Figure 2.**
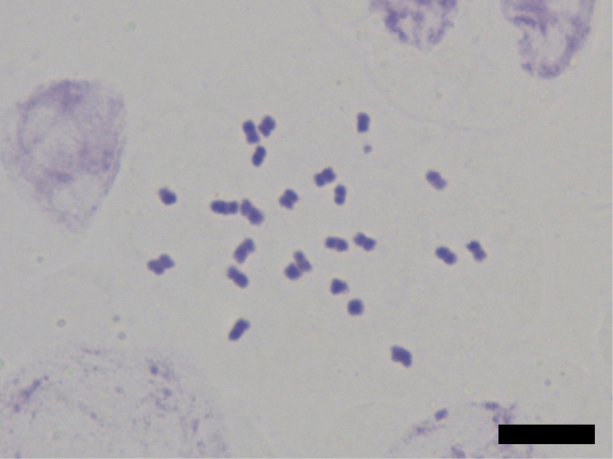
Mitotic metaphase chromosomes from the root tip of *Orychophragmus yangii*. Scale bar: 10 μm.

Five root tips from each of the two populations of the new species were observed. For each root tip, at least two cells with well-spread chromosomes were counted. Results show that all cells have the chromosome number of 2*n* = 24 (Fig. 2).

## ﻿Taxonomy

### ﻿ Orychophragmus yangii

Taxon classificationPlantaeBrassicalesBrassicaceae

L.L.Xun & M.Yue sp. nov.

4219BE73-70C8-534B-B059-FA81C1CE49B6

urn:lsid:ipni.org:names:77368775-1

Figs 3, 4

#### Type.

China • Shaanxi Province, Suide County, on arid areas of sunny slope, 830 m a.s.l., 37°29'50.10"N, 110°15'42.06"E, 18 Apr. 2023, *L. L. Xun 3061* (holotype XBGH!; isotypes XBGH!, WUK!).

#### Diagnosis.

The new species is different from all other *Orychophragmus* species by the following traits: whole plant glabrous; leaves vary from simple to pinnatifid but never lyrate; the pinnatifid leaves have lobes divided or not; the simple leaves are widest at base; sepals green; petals white; fruit narrowly linear, terete, glabrous; rostra of fruit 2–4 mm long.

#### Description.

Herbs annual or biennial. Stems erect or ascending, 12–50 cm tall, simple or branched, glabrous. Basal and lower cauline leaves pinnatifid, lateral lobes 1–10 on each side, lobes divided or not, margin entire; petiole 1–7 cm long; blade oblong-ovate, 2.5–13 × 1–5 cm, glabrous; often withered when fruiting. Middle and upper cauline leaves pinnatifid or simple, amplexicaul, sessile, margin entire, apex acute or acuminate, oblong-ovate, 2–20 × 0.5–6 cm; lateral lobes (if present) 1–7 on each side. Raceme ebracteate, erect. Sepals 4, green, linear, erect, 8–14 × 1–2 mm, glabrous, base of lateral pair strongly saccate. Petals 4, white, obovate, 8–15 × 5–8 mm, apex rounded; claw as long as sepals. Filaments 6, 0.6–1.1 cm; anthers linear, 3–4 mm, apiculate, erect or recurved. Ovary linear, ovules many, style 1–2 mm. Pedicels glabrous, 0.8–1.5 cm long. Fruit narrowly linear, terete, (2-) 4–10 cm × 1.5–2.0 mm, rostra short, 2–4 mm long; valves glabrous. Seeds dark brown, oblong, 1.7–2.5 × 0.6–1.0 mm, surface with reticulation.

**Figure 3.**
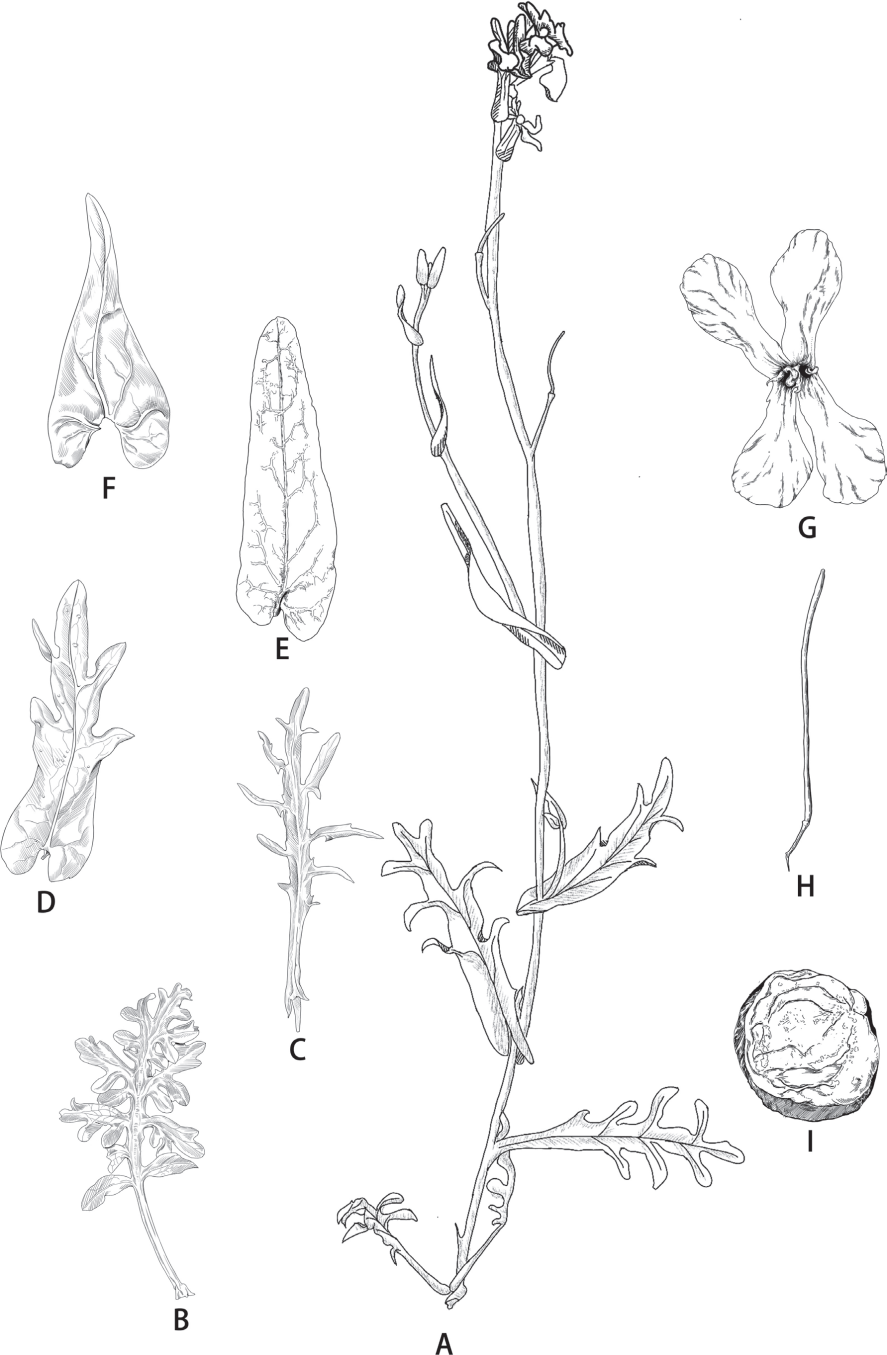
Illustration of *Orychophragmus yangii.* A. Plant; B–F. Leaves; G. Flower; H. Fruit; I. Transverse section of fruit. Drawn by Jia-Chen Liang.

#### Phenology.

Flowering from April to May, fruiting from May to June.

#### Distribution, habitat, and ecology.

The distribution of *Orychophragmus yangii* is limited to Suide and Wubu County of Shaanxi Province. It can be considered endemic to the Loess Plateau and Shaanxi Province. This species usually grows on the dry and sunny slope, roof, and roadside from 800 M to 900 m a.s.l.

**Figure 4.**
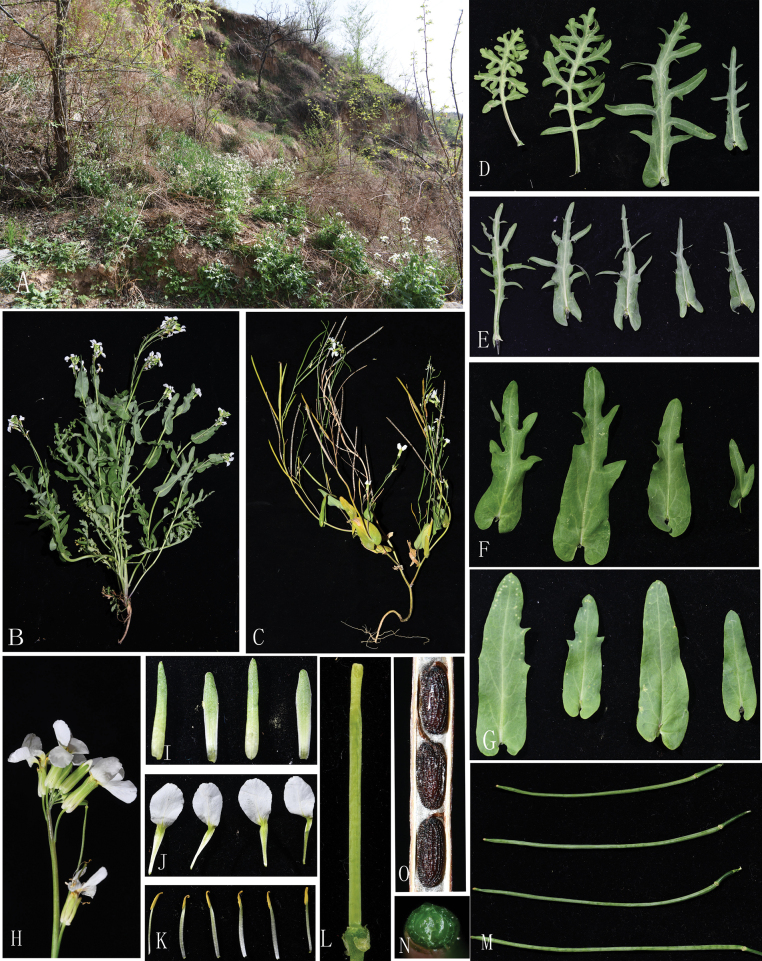
The habitat and morphological characters of *Orychophragmus yangii*. A. Habitat; B. Plants in bloom; C. Fruiting plants; D–G. Leaves; H. Inflorescences; I. Sepals; J. Petals; K. Stamens; L. Pistil; M. Fruits; N. Transverse section of fruit; O. Seeds.

#### Etymology.

The specific epithet “*yangii*” is derived from Mr. Hong-Hua Yang. The Chinese vernacular name of this new species can be “宏华诸葛菜 (hóng huá zhū gě cài)”.

#### Other specimens checked (Paratypes).

China • Shaanxi Province, Suide County, on the hillside, ca. 900 M, 14 May 1956, *Huanghedui 6839* (WUK!); • Suide County, on the sunny place near Long Bay, 7 Jun 1953, *K. T. Fu 6678* (HIB!); • Suide County, 19 May 2021, *L. L. Xun 1564* (XBGH!); • Suide County, on dry roof, ca. 810 M, 37°29'56.2"N, 110°15'32.97"E, 13 June 2021, *L. L. Xun 1650* (XBGH!); • Suide County, on the sunny place of roadside, ca. 800 M, 37°29'57.7"N, 110°15'29.2"E, 18 April 2023, *L. L. Xun 3059* (XBGH!); • Wubu County, on the sunny place of loess hill, ca. 800 M, 23 May 1953, *Shanbeidui* 10070 (PE!).

## ﻿Discussion

The new species *O. yangii* is very distinctive in the genus *Orychophragmus*. The lower leaves of the new species are pinnatifid, never lyrate, with lobes divided or not, whereas the leaves of other species are lyrate—rarely pinnatifid in *O. violaceus*. The petals of *O. yangii* are exclusively white, while the other seven species of *Orychophragmus* have predominantly purple or lavender petals; only rarely do some individuals of *O. violaceus* have white petals. The fruit rostra of *O. yangii* are clearly shorter than those of other *Orychophragmus* members. Ecologically, *O. yangii* grows in arid areas in sunny locations of the Loess Plateau, which differs markedly from other species that grow in more humid conditions in sunny or shady locations. Such distinctive morphological and ecological characters of the new species coincide with its phylogenetic isolation from other *Orychophragmus* species. It is likely that *O. yangii* split early in the evolution of *Orychophragmus* and adapted to a different environment with distinct morphological traits compared with other species in the genus.

A detailed morphological and ecological comparison of the new species with the most widespread *O. violaceus* is provided in Table 1.

**Table 1.** Comparison of *Orychophragmus yangii* with *O. violaceus*.

Characters	O. yangii	O. violaceus
Leaves	Vary from simple to pinnatifid but never lyrate; the pinnatifid leaves have lobes divided or not; the simple leaves are widest at base; margin entire.	Simple, lyrate, rarely pinnatifid; lobes rarely divide; the simple leaves are widest at center; margin dentate, rarely entire.
Petals	White	Deep purple or lavender, rarely white
Fruits	Terete, glabrous; rostra 2–4 mm long.	Four-angled, glabrous or densely hirsute; rostra 15–25 mm long.
Seeds	1.7–2.5 × 0.6–1.0 mm	3.0–4.0 × 1.2–1.7 mm
Habitat	Sunny, arid place	Sunny or shaddy, humid place

## Supplementary Material

XML Treatment for Orychophragmus yangii

## ﻿Additional information

### Use of AI

No use of AI was reported.

### Author ORCIDs

ALu-Lu Xun https://orcid.org/0000-0002-6861-5883

Yong-Xing Qin https://orcid.org//0009-0004-4562-3460

Pei-Liang Liu https://orcid.org//0000-0001-6566-7824

Ming Yue https://orcid.org/0000-0002-8618-1126

Bin Li https://orcid.org/0000-0001-9254-8003

Yong-Fu Chai https://orcid.org/0000-0001-8694-2629

### Data availability

The newly generated DNA sequences have been deposited in GenBank, and the accession numbers are provided in Appendix 1.

## ﻿Appendix 1

Taxon name, geographical locality, voucher, herbarium code, and GenBank accession number for the sequences used in this study. For each sample, accession numbers are given for the plastid genome and the nuclear sequences. New sequences generated in this study are indicated by an asterisk (*).

*Aethionema grandiflorum* Boiss. & Hohen.: NC_009266, SRR14574152;

*Arabidopsis thaliana* (L.) Heynh.: AP000423, SRR33449606;

*Brassica juncea* (L.) Czern. & Coss.: KT581449, KX709362;

*Brassica napus* L.: GQ861354, KX709366;

*Capsella grandiflora* (Fauché & Chaub.) Boiss.: KR029092, SRR8904467;

*Carica papaya* L.: EU431223, SRR31677748;

*Eutrema heterophyllum* (W. W. Sm.) H. Hara: KT270358, SRR6754263;

*Lobularia maritima* (L.) Desv.: NC_009274, SRR11695922;

*Orychophragmus violaceus* (L.) O. E. Schulz: KX364399, MK637769, SRR18885249;

*Orychophragmus yangii* L. L. Xun & M. Yue: China, Shaanxi, Suide county, L. L. Xun 3061 (XBGH and WUK), PX116179*, PX111728*; L. L. Xun 1650 (XBGH), PX116180*, PX111729*;

*Orychophragmus hupehensis* (Pamp.) Z. M. Tan & X. Liang Zhang: KX756551;

*Orychophragmus longisiliquus* Huan Hu et al.: KX756549, SRR6655848;

*Orychophragmus taibaiensis* Z. M. Tan & B. X. Zhao: China, Shaanxi, Taibai, Mt. Taibai, L. L. Xun 2401, KX756550, PX111730*;

*Orychophragmus zhongtiaoshanus* Huan Hu et al.: KX756547, SRR6655838;

*Orychophragmus diffusus* Z. M. Tan & J. M. Xu: KX756548, SRR6655830;

*Sinalliaria limprichtiana* (Pax) X. F. Jin et al.: KX342848, SRR6441719;

Sinalliaria limprichtiana var. grandifolia (C. H. An) X. F. Jin et al.: KX342847, SRR6441715.
